# Effect of Interventions With a Clinical Decision Support System for Hospitalized Older Patients: Systematic Review Mapping Implementation and Design Factors

**DOI:** 10.2196/28023

**Published:** 2021-07-16

**Authors:** Birgit A Damoiseaux-Volman, Nathalie van der Velde, Sil G Ruige, Johannes A Romijn, Ameen Abu-Hanna, Stephanie Medlock

**Affiliations:** 1 Department of Medical Informatics Amsterdam Public Health Research Institute Amsterdam UMC, University of Amsterdam Amsterdam Netherlands; 2 Section of Geriatric Medicine Amsterdam Public Health Research Institute Amsterdam UMC, University of Amsterdam Amsterdam Netherlands; 3 Department of Medicine Amsterdam Public Health Research Institute Amsterdam UMC, University of Amsterdam Amsterdam Netherlands

**Keywords:** aged, clinical decision support systems, geriatrics, hospital, quality of care

## Abstract

**Background:**

Clinical decision support systems (CDSSs) form an implementation strategy that can facilitate and support health care professionals in the care of older hospitalized patients.

**Objective:**

Our study aims to systematically review the effects of CDSS interventions in older hospitalized patients. As a secondary aim, we aim to summarize the implementation and design factors described in effective and ineffective interventions and identify gaps in the current literature.

**Methods:**

We conducted a systematic review with a search strategy combining the categories *older patients*, *geriatric topic*, *hospital*, *CDSS*, and *intervention* in the databases MEDLINE, Embase, and SCOPUS. We included controlled studies, extracted data of all reported outcomes, and potentially beneficial design and implementation factors. We structured these factors using the Grol and Wensing Implementation of Change model, the GUIDES (Guideline Implementation with Decision Support) checklist, and the two-stream model. The risk of bias of the included studies was assessed using the Cochrane Collaboration’s Effective Practice and Organisation of Care risk of bias approach.

**Results:**

Our systematic review included 18 interventions, of which 13 (72%) were effective in improving care. Among these interventions, 8 (6 effective) focused on medication review, 8 (6 effective) on delirium, 7 (4 effective) on falls, 5 (4 effective) on functional decline, 4 (3 effective) on discharge or aftercare, and 2 (0 effective) on pressure ulcers. In 77% (10/13) effective interventions, the effect was based on process-related outcomes, in 15% (2/13) interventions on both process- and patient-related outcomes, and in 8% (1/13) interventions on patient-related outcomes. The following implementation and design factors were potentially associated with effectiveness: *a priori problem or performance analyses* (described in 9/13, 69% effective vs 0/5, 0% ineffective interventions), *multifaceted interventions* (8/13, 62% vs 1/5, 20%), and *consideration of the workflow* (9/13, 69% vs 1/5, 20%).

**Conclusions:**

CDSS interventions can improve the hospital care of older patients, mostly on process-related outcomes. We identified 2 implementation factors and 1 design factor that were reported more frequently in articles on effective interventions. More studies with strong designs are needed to measure the effect of CDSS on relevant patient-related outcomes, investigate personalized (data-driven) interventions, and quantify the impact of implementation and design factors on CDSS effectiveness.

**Trial Registration:**

PROSPERO (International Prospective Register of Systematic Reviews): CRD42019124470; https://www.crd.york.ac.uk/prospero/display_record.php?RecordID=124470.

## Introduction

### Background

In hospitals, the number and proportion of older patients have increased in the past years and will continue to grow in the following years [1,2]. Hospitalization has a significant impact on the lives of older patients. The incidence of preventable adverse events in a hospital setting is almost twice as high in older patients as in younger patients [3]. In addition, there is a high prevalence of geriatric syndromes and a high risk of functional decline and mortality in older hospitalized patients [4,5]. Geriatric syndromes are described as “common, serious conditions for older persons, holding substantial implications for functioning and quality of life” [6]. In a representative cohort investigating geriatric syndromes in older patients from 3 acute care hospitals, the prevalence of bladder incontinence was 37%, 5% for pressure ulcers, and 18% for delirium [4]. Furthermore, 6% of the patients suffered from one or more falls during the hospital stay [4]. Geriatric syndromes, involvement of multiple health care professionals, and difficulties in communicating with patients complicate hospital care.

Clinical decision support systems (CDSSs) can facilitate and support health professionals in the complex care of older hospitalized patients. CDSSs have the potential to transfer knowledge from guidelines to physicians, pharmacists, and nurses or experts to all hospital physicians, for example, from geriatricians to other specialties. Furthermore, CDSSs can support the implementation of advice in hospital practice by structuring information from different departments or performing calculations [7]. Our previous work indicated that there are several areas where a CDSS is perceived as having the potential to improve geriatric care in the hospital, including falls and delirium [8]. To date, systematic reviews of CDSS for the care of older patients have focused solely on medication and not on other aspects of care [9-11].

Systematic reviews of CDSS interventions, not specifically for older patients, have identified factors that could be associated with CDSS effectiveness, such as providing patient-specific advice [12,13]. Evidence for these factors is low, and further trials are needed to conclude which factors improve effectiveness [13]. A CDSS supporting health care professionals in geriatric care may differ and be more difficult to design and implement because of the complexity of care and the need for hospital-wide interventions. However, the implementation and design factors influencing the effect of CDSS interventions to improve geriatric care have not been studied in a systematic review.

### Objectives

Our study aims to systematically review the effect of CDSS interventions on common problems in the care of older hospitalized patients. The secondary aim is to summarize the implementation and design factors described in the effective or ineffective interventions and identify gaps in the current literature.

## Methods

### Protocol

The protocol of our systematic review was registered and published on the website of the PROSPERO (International Prospective Register of Systematic Reviews) with the registration number CRD42019124470. Multimedia Appendix 1 contains the completed PRISMA (Preferred Reporting Items for Systematic Reviews and Meta-Analyses) checklist [14].

### Search Strategy

A search strategy combining the categories *older patients*, *geriatric topic*, *hospital*, *CDSS*, and *intervention* was designed and adapted for the databases MEDLINE (via Ovid), Embase (via Ovid), and SCOPUS. The search strategy was based on keywords, medical subject headings, and text words. The search was conducted until April 15, 2020. The full search strategy is shown in Multimedia Appendix 2. Duplicates in the search were detected and deleted in EndNote X9 (Clarivate Analytics), 2019 [15]. In addition, we screened the references of the included studies for missing articles.

### Study Selection

Using a checklist with prespecified eligibility criteria, 2 researchers (BADV and SGR) screened articles for inclusion. These criteria were piloted in the first 200 articles and subsequently adjusted, if necessary. Title and abstract screening was performed using Rayyan [16]. The eligibility criteria were (1) intervention with CDSS, (2) geriatric topic in the care of hospitalized patients aged 65 years or older, (3) evaluation in a controlled trial (including before-after and other quasi-experimental designs), and (4) peer-reviewed journal paper in English. We required that the eligibility criteria were met on the basis of the abstract.

For CDSS, we used the definition of Musen et al [17] of “any computer program designed to help health care professionals to make clinical decisions.” The geriatric topics were derived from our previous study [8], in which we determined which areas of geriatric care CDSS can potentially improve the care of hospitalized older patients and, in addition, the work of Inouye et al [6] describing 5 common geriatric syndromes. The topics included were pressure ulcers, incontinence, falls, functional decline, delirium, medication review, communication with the patient (at discharge), planning (in the hospital), and (communication and collaboration between health care professionals at) discharge and aftercare. For medication review, we used the definition of the Pharmaceutical Care Network Europe, “Medication review is a structured evaluation of a patient’s medicines with the aim of optimising medicines use and improving health outcomes.” This definition entails detecting drug-related problems and recommending interventions [18]. The geriatric topics had to be part of the inclusion criteria, the aim, or the outcomes of the study.

### Data Extraction and Risk of Bias Assessment

#### Overview

Two researchers (BADV and SGR) individually conducted data extraction and risk of bias assessment. We used a data extraction form for data extraction. The form was tested on 2 papers and adjusted as required. If an article referred to another article describing the development or implementation of the intervention, data from this additional article were also extracted. The risk of bias of the included studies was assessed using Cochrane Collaboration’s Effective Practice and Organisation of Care (EPOC) risk of bias approach [19]. We extracted all reported outcomes from the included articles: process-related, patient-related, and cost outcomes. Patient-related outcomes could be either clinical or patient-derived outcomes [20]. We extracted data on outcomes measured in both the control and intervention groups. Each step of the inclusion process—data extraction, structuring and mapping of the implementation and design factors, and risk of bias assessment—was conducted independently by 2 researchers (BADV and SGR), and the results were compared. Disagreements were discussed until agreement was achieved and, if necessary, resolved by a third researcher (SM).

#### Effectiveness of the Interventions

We used a definition of effectiveness that was previously used in the literature [12]. Interventions were considered effective when the prespecified primary outcome, ≥50% of the prespecified primary outcomes, or, if a primary outcome was not defined, ≥50% of the prespecified outcomes showed significant (*P*<.05) improvement [12]. If an intervention was described in more than one article, the outcomes from all articles assessing the intervention were used to define the effectiveness.

#### Implementation and Design Factors

We extracted data on implementation and design factors. The implementation factors were classified according to the Grol and Wensing Implementation of Change model [21]. Implementation is defined as “a planned process and systematic introduction of innovations and/or changes of proven value” [21]. The model describes the steps for improving patient care with an intervention and summarizes the implementation literature. We extracted any activities that the authors described, which fit one or more steps in this model. Step 4 in this model is the selection of an implementation strategy. To define implementation strategies, we used the classification of implementation strategies in the EPOC taxonomy [22]. Implementation strategies (such as a CDSS or audit and feedback) that fit into the EPOC classification were also extracted from the included studies.

Design factors were classified according to the GUIDES (Guideline Implementation with Decision Support) checklist and the two-stream model [23,24]. The GUIDES checklist is a tool to support the development of successful CDSS and describes 4 groups: content, context, system, and implementation of the CDSS (eg, appropriateness of the information about CDSSs to users). The two-stream model contains elements describing factors that can potentially influence the success of a CDSS. We categorized the two-stream model elements into the 4 groups of the GUIDES checklist to obtain a complete picture of the potential design factors.

#### Data Synthesis

We conducted a narrative synthesis and counted which implementation and design factors were described in more effective interventions than ineffective interventions.

## Results

### Search Results

**Figure 1:**
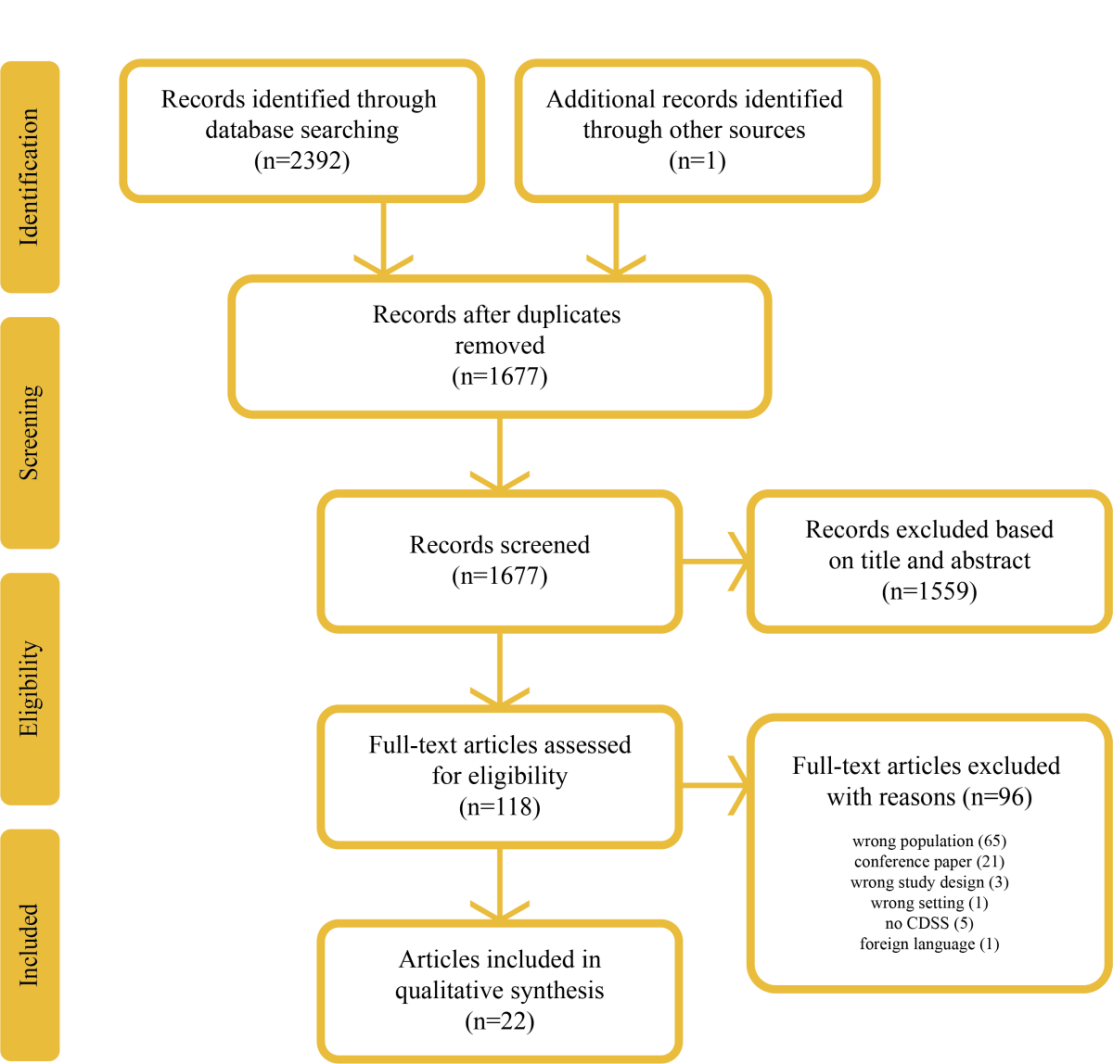
PRISMA (Preferred Reporting Items for Systematic Reviews and Meta-Analyses) flow diagram of search results. CDSS: Clinical Decision Support Systems.

A total of 2392 articles were identified in the search. Figure 1 shows the PRISMA flow diagram with the number of articles excluded after each screening step and the reasons for excluding the full-text articles. A total of 22 articles were eligible for inclusion in our systematic review.

### Characteristics of Included Studies

All the characteristics of the included articles are shown in Multimedia Appendix 3 [25-46]. The 22 articles described interventions performed in 5 countries: 12 studies in the United States, 5 in Canada, 3 in Ireland, 1 in Italy, and 1 in France.

In total, 18 different CDSS interventions were described in the 22 included articles (Multimedia Appendix 4). A CDSS intervention was described in 3 articles: 1 article compared prescriptions at admission and discharge of the intervention group, 1 article described the main randomized controlled trial (RCT), and 1 article described the cost-effectiveness of the RCT [25-27]. Another CDSS intervention was described in 2 articles: 1 article evaluated the implementation at the initial site, and 1 article evaluated the implementation at 4 sites [28,29]. Finally, 1 CDSS intervention was linked in 2 articles: 1 article described a subgroup analysis of the earlier RCT [30,31].

Different study designs were selected to evaluate the interventions; 1 article used a cluster-randomized study, 7 articles used an RCT design, 1 article used a stepped wedge trial design, 2 articles used an interrupted time series design, and 11 articles used a before-after design. All RCTs had a registration of a protocol [27,30-34].

### Risk of Bias Assessment

Multimedia Appendix 5 [25-46] shows the results of the risk of bias assessment. In 4 of the 22 articles, all suggested risk of bias criteria were categorized as low or unclear [32,35-37]. Other articles had 1 or more high risks for bias [25-31,33,34,38-46]. We did not find descriptions of the amount of missing data or how missing data were handled in any of the articles. All 7 RCTs had a high or unclear risk for protection against contamination [27,30-34]. The most frequent source of bias was “flawed or absent random sequence generation,” present in 14 studies [25-29,38-46]. This was mainly because of studies with a nonrandomized design (eg, before-after studies).

### Effectiveness, Outcomes, and Geriatric Topic

In total, 72% (13/18) of interventions were effective in improving care, mainly with regard to process-related outcomes [25,27-29,32,34,35,37,39-42,44-46]. In 77% (10/13) of effective interventions, the effect was based on process-related outcomes, in 15% (2/13) of interventions on both process and patient-related outcomes, and in 8% (1/13) interventions on patient-related outcomes. In 60% (3/5) ineffective interventions, the results were based on both process and patient-related outcomes, in 20% (1/5) of interventions on patient-related outcomes and in 20% (1/5) interventions significance was not calculated; according to the definition we adopted in our review, this intervention was considered ineffective.

Of the 18 interventions, 8 (44%; 6 effective) focused on medication review, 8 (44%; 6 effective) on delirium, 7 (39%; 4 effective) on falls, 5 (28%; 4 effective) on functional decline, 4 (22%; 3 effective) on discharge or aftercare, and 2 (11%; 0 effective) on pressure ulcers. None of the interventions focused on incontinence, planning, or communication with patients at discharge. Part of the interventions on falls (3/7, 43%) and delirium (3/8, 38%) focused on improving drug prescription and not on other risk factors. For discharge, 2 of 4 interventions focused on (and succeeded in) improving prescriptions at emergency department discharge [28,29,32].

We grouped the 81 different outcomes into 6 groups: medication (35), location or duration (11), prevention of geriatric conditions (20), prevalence of geriatric conditions (10), survival (3), and costs (2). Outcomes in the medication and prevention of geriatric conditions groups were mostly process-related. Outcomes in the groups of prevalence of geriatric conditions and survival were mostly patient-related.

Patient-related outcome length of stay was measured in 10 interventions, none of which were primary outcomes, and none of them showed a significant improvement [26,30,31,34,36,38-44]. The 5 interventions measuring 30-day readmission also failed to show an effect on this outcome [30,33,34,39,43]. Other outcomes that did not show an effect in the included studies were survival and cost outcomes, delirium, and orders for consultation [26,30,31,34,36,39-41].

Patient-related outcomes that showed a statistically significant improvement (*P*=.04) were falls, adverse drug reactions, and discharged home (percentage of patients who went home after discharge). Falls or fall rates were measured in 6 interventions and significantly reduced in 2 (primary outcome in 1) [30,36-38,41,42]. Adverse drug reactions or adverse drug events were measured in 2 interventions and significantly reduced in 1 (primary outcome) [26,27,45]. Discharged home was measured in 2 interventions and significantly improved in 1 (no primary outcomes) [30,31,39].

### Implementation Factors

Articles about effective interventions described more often an *a priori problem or performance analyses* and/or included more often *multifaceted interventions* than articles about ineffective interventions. As Multimedia Appendix 4 shows, in 69% (9/13) effective interventions and 0% (0/5) ineffective interventions, *a priori problem or performance analyses* were conducted before implementation [28,29,32,34,35,37,39,40,44,45]. This was done by reviewing prescribing data, investigating barriers and facilitators, mapping the use of computerized physician order entry, or describing care before implementation. In total, 62% (8/13) effective interventions and 20% (1/5) ineffective interventions were *multifaceted interventions* implying that the intervention had more than one implementation strategy [25-29,34,35,39-41,43,44].

Multimedia Appendix 6 [25-46] shows all implementation and design factors per included article based on the Grol and Wensing Implementation of Change model, the GUIDES checklist, and the two-stream model. None of the included interventions described all 7 steps of the Grol and Wensing Implementation of Change model. All interventions reported an implementation strategy (step 4 in the model). All interventions described a CDSS, which is included in the implementation strategy *reminder*. Aside from *reminder*, the multifaceted interventions used varying strategies: 8 interventions described an educational strategy (7 effective), 2 audit and feedback (2 effective), 2 practice and setting (2 effective), 2 organizational culture (1 effective), and 1 local consensus processes (1 effective).

### CDSS Design Factors

Articles of effective interventions described only 1 design factor more frequently than articles of ineffective interventions: *consideration of the workflow*. The workflow before implementation was described or considered in the CDSS development in 69% (9/13) effective interventions and 20% (1/5) ineffective interventions [25-29,32,36,37,39-42].

The other design factors are shown in Multimedia Appendix 6. Almost all studies described the clinical knowledge of CDSS. None of the studies described clinical knowledge based on prediction models or machine learning. Clinical knowledge was mostly based on the Beers criteria, STOPP (Screening Tool of Older Persons’ Prescriptions)/START (Screening Tool to Alert to Right Treatment) criteria, experts, guidelines, or scientific literature [47-51]. In 11 interventions (8 effective), a multidisciplinary team with geriatricians and pharmacists was involved in selecting the clinical knowledge of the CDSS [25-29,32-35,40,42,43,45].

Overall, the presentation of the CDSSs varied and included 6 patient-specific reports (4 effective), 1 in-basket message (0 effective), 7 (non) interruptive alerts (5 effective), 2 default doses in computerized physician order entry (2 effective), and 6 (dynamic) order sets (5 effective). Only 5 interventions, of which 2 were effective, described the use of patient data from multiple parts of the patient record or multiple sources [33,34,43,45,46]. For medication review, 6 of 8 interventions described CDSSs built as stand-alone systems and therefore not integrated into the electronic health record [25-27,34,35,38,45,46]. The users of the systems were physicians in 9 interventions (7 effective), pharmacists in 6 interventions (5 effective), and nurses in 4 interventions (3 effective). Only 3 studies described a CDSS for multiple specialists [40,43-45].

## Discussion

### Principal Findings

In our systematic review, we found 22 articles describing 18 different CDSS interventions for the care of older hospitalized patients evaluated in controlled trials (including before-after and other quasi-experimental designs). These CDSS interventions focused on medication review, falls, delirium, discharge or aftercare, functional decline, and pressure ulcers. In total, 72% (13/18) of the included CDSS interventions effectively improved geriatric care, mainly concerning process-related outcomes. Two implementation factors—*a priori problem or performance analyses* and *multifaceted interventions*—and 1 design factor—*consideration of the workflow*—were described in more articles of effective interventions than ineffective ones. These factors are potentially associated with effectiveness; however, more trials are needed to quantify their impact or assess whether this association is causal in nature. No factors potentially associated with ineffectiveness were identified. We did not find any CDSS interventions for three geriatric problems: incontinence, planning, or communication with patients at discharge. The included interventions had limited effectiveness on patient outcomes. Furthermore, we found no data-driven CDSS in our systematic review.

Most of the 18 included interventions focused on medication review, delirium, and falls. We did not find any CDSS interventions for incontinence, planning, or communication with patients at discharge, and none of the CDSS interventions effectively improved care for pressure ulcers. Of the 8 interventions on medication review, 6 (75%) showed an improvement in prescribing for geriatric patients. This finding aligns with previous systematic reviews, which also stated that computerized support could improve prescribing for older patients [9-11]. For delirium and falls, 75% (6/8) of CDSS interventions improved care for delirium and 57% (4/7) for falls. Our review is the first to assess the effect of CDSS interventions on these common geriatric syndromes in older patients. Notably, even though these geriatric syndromes are multifactorial, almost half of the interventions for falls and delirium addressed only a single risk factor.

We found only 3 factors—2 implementation factors and 1 design factor, which were described in more articles about effective interventions than ineffective ones. In contrast to previously published reviews, no other design factors were identified in our study [12,13]. This could be because of the relatively small number of published CDSS interventions assessing the effect on geriatric care in a controlled trial; 2 of the 3 factors identified in our review were described in previous literature. In line with best practices in implementation science, *a priori analysis of problems and actual performance* was described more often in studies with positive outcomes [21]. The second approach, incorporating CDSS within the workflow, is in accordance with best practices as well [52-54]. However, for the third factor, the literature is inconsistent. We found a potential positive effect of multifaceted interventions. In the implementation science literature, it is not clear whether multifaceted interventions are more effective than single interventions [55]. For falls, previously published systematic reviews also showed inconsistent results from multifaceted interventions, not specifically with CDSS, in hospitals [56,57].

Scientific literature in geriatrics often has a lower level of evidence because of heterogeneous patient characteristics and the underrepresentation of older patients in clinical trials [58]. Consequently, the clinical knowledge underlying CDSS has a lower level of evidence. The quality of clinical knowledge is important for the impact of the CDSS [59]. For the uptake and acceptance of CDSS in geriatric care, evaluation studies would preferably include patient outcomes not only to contribute to evidence on the effectiveness of the system but also to contribute evidence for the clinical knowledge. Our results showed that patient-related outcomes rarely significantly improved. This can be partly explained by the fact that only 3 interventions were evaluated with a patient-related outcome as the primary outcome, study sample sizes were too small to assess patient outcomes, and/or the choice of patient-related outcomes. In our systematic review, general patient-related outcomes such as length of stay and 30-day readmission did not improve; however, specific patient-related outcomes such as falls and adverse drug events were improved in some of the studies. A paper describing a framework for study designs in patent safety science stated that a common problem is that general patient-related outcomes can be influenced by factors other than the intervention [20]. Other systematic reviews of CDSSs also found sparse evidence for the association of CDSS with patient outcomes [9,12,60,61]. Two systematic reviews mentioned possible reasons: short duration of studies and logistics difficulties measuring the direct effect on patient outcomes and conducting RCTs for CDSS interventions [12,61]. On the contrary, a systematic review of CDSS for inpatients did find an effect on patient-related outcomes [59]. Future studies in geriatric CDSS should include a large enough sample size and duration and select appropriate outcomes directly influenced by the intervention to show significant effects on patient-related outcomes.

In our review, none of the clinical knowledge of the included CDSSs was data-driven; for example, it was based on prediction models or machine learning. Data-driven methods typically analyze large and complex data sets and are promising for CDSS [62,63]. However, evidence of the effectiveness of data-driven CDSS is thus far limited [63]. Challenges for data-driven CDSS include having the models as *black boxes that hamper users’* understanding of the clinical knowledge underlying CDSS [62]. An example of an effective data-driven CDSS without a *black box* is described in the study by Cho et al [64]. In this study, not specifically focused on older patients and therefore not included in our systematic review, a CDSS for pressure ulcers was developed with a Bayesian Network model and linked to the hospital electronic health record. The CDSS effectively reduced the prevalence of pressure ulcers and intensive care unit length of stay [64]. More studies are needed to explore the possibilities of data-driven CDSS for complex populations, such as older hospitalized patients.

The EPOC tool was used to assess the risk of bias in all studies. Nonrandomized study designs (eg, before-after studies) already have a high risk of bias because of their study design. Therefore, the overall bias of the included studies was high, except for 4 studies. Future evaluation studies should use randomized designs where possible or high-quality, nonrandomized designs, such as time series.

### Strengths and Limitations

Our systematic review is the first to provide an overview of the effect of CDSSs in improving care for various common geriatric problems in hospital care for older patients. It is complementary to previously published articles on CDSS for prescribing in this population [9]. CDSSs targeting aspects of care other than medication have not been previously studied in a systematic review. A strength of our study is that we incorporated implementation and design factors in the analysis to contribute to the understanding of CDSS effectiveness in this population. We used previous literature on geriatric care, implementation science, and CDSS to select geriatric topics and structure the implementation and design factors. Another strength of this study is that we used a broad and comprehensive search strategy, including checking the references of the studies. We chose to include all controlled studies; both RCTs and quasi-experimental studies. RCTs are generally considered the highest level of evidence; however, an RCT is often not practical in a CDSS implementation because of contamination issues. Thus, our choice to include other study designs provides a more representative picture of studies conducted with CDSSs.

A limitation of our study is that the included studies and extracted outcomes are heterogeneous and, therefore, not sufficiently comparable for quantitative analysis. More intervention studies are needed to quantify the effects on specific geriatric problems and investigate potential influencing factors on the effectiveness of these CDSS interventions. Implementation and design factors not described in the articles were not included in the analysis, which may have led to the underrepresentation of these factors. Furthermore, 2 of the 18 included CDSS interventions used almost the same implementation strategy in the same hospital, but at different periods and with a different CDSS design: the first intervention had a manual entry and the second was automatic [34,35]. Our results can be affected by publication bias because, especially with weaker study designs, studies showing an effect are more likely to be published. The inclusion and data extraction processes were performed by 2 individual researchers to minimize potential bias.

### Conclusions

In conclusion, our systematic review shows that CDSS interventions have the potential to improve the hospital care of older patients. In total, 72% (13/18) of the included interventions were effective (mostly on process outcomes). Two implementation factors—*a priori problem or performance analyses* and *multifaceted interventions*—and 1 design factor—*consideration of the workflow*—were reported more frequently in articles of effective interventions. However, more studies are needed to assess the impact of a CDSS intervention on care for older hospitalized patients. Future studies should use a strong study design, such as a randomized trial or interrupted time series. RCTs are often challenging in CDSS research because of the risk of contamination and technical issues in randomizing the intervention. Furthermore, future studies should include a large enough sample size and duration and select specific patient-related outcomes directly affected by the intervention. Future studies should assess the effect on geriatric conditions, quantify the impact of implementation and design factors on CDSS effectiveness, and investigate the potential of personalized (data-driven) interventions.

## Appendix

Multimedia Appendix 1 PRISMA (Preferred Reporting Items for Systematic Reviews and Meta-Analyses) checklist.

Multimedia Appendix 2 Search strategy.

Multimedia Appendix 3 Study design, characteristics, and outcomes of the included studies.

Multimedia Appendix 4 Study design, characteristics, outcomes, and main implementation or design factors of the included articles.

Multimedia Appendix 5 Risk of bias.

Multimedia Appendix 6 Implementation and design factors described in the included studies.

## Abbreviations

CDSS: clinical decision support system
EPOC: Effective Practice and Organisation of Care
GUIDES: Guideline Implementation with Decision Support
PRISMA: Preferred Reporting Items for Systematic Reviews and Meta-Analyses
PROSPERO: International Prospective Register of Systematic Reviews
RCT: randomized controlled trial
START: Screening Tool to Alert to Right Treatment
STOPP: Screening Tool of Older Persons’ Prescriptions
