# Supplementary material for: Effect of Interventions With a Clinical Decision Support System for Hospitalized Older Patients: Systematic Review Mapping Implementation and Design Factors
Source: JMIR Med Inform. 2021 Jul 16;9(7):e28023. doi: 10.2196/28023 (PMC8325084; doi:10.2196/28023)
Supplement: Multimedia Appendix 2 [file medinform_v9i7e28023_app2.docx]

**Appendix 2: Search strategy**

Embase

((exp aging/ or exp aged/ or elderly care/ or geriatric care/ or cognitive dysfunction/ or multimorbidity/ or (older person? or older patient? or (senior* not school) or elder or elders or elderly or older adult* or geriatric* or cognitive dysfunction or multimorbidity or frailty or postmenopausal women or community-dwelling or (old* adj3 people) or (old* adj3 person?) or (old* adj3 patient?) or (old* adj3 client?)).ab,kw,ti. or (older m?n or older wom?n).ab,kw,ti. or (("65" or "70" or "75" or "80") adj2 (year? or yr* or age?)).ab,kw,ti. or (geriatr* or age or aging or elderl* or gerontolog*).jx)

AND

(falling/ or urinary Incontinence/ or delirium/ or *polypharmacy/ or medication therapy management/ or geriatric assessment/ or hospital planning/ or hospital discharge/ or (fall? or fell or falling or fallen or faller* or functional decline or delirium or urinary Incontinence or pressure ulcer* or polypharmacy or (review adj3 (drug? or medication)) or inappropriate medication* or medication reconciliation or medication changes or planning or geriatric assessment or discharge letter or discharge planning or discharge communication or (manag* adj3 therapy)).ab,kw,ti.))

AND

(exp hospital/ or exp hospital management/ or hospital patient/ or aged hospital patient/ or exp hospitalization/ or hospital care/ or (inpatient* or department of veterans affairs or ICU* or intensive care unit* or specialist* or specialty or acute aged care setting or secondary care or tertiary care or ward* or hospital* or medical center*).ab,kw,ti.)

AND

(decision support system/ or ("decision support" or DSS or CDSS or CDS or CCDS or CCDSS or computer-generated or decision making support or expert system* or ((computer* or software or electronic or automat* or IT system* or information technology) and (tailor* or advice or advise or warning* or prompt* or remind* or alert* or algorithm* or tool* or order entry or order set*)) or computer* dos*3 or electronic dos*3 or automat* dos*3 or dos*3 calculator).ab,kw,ti.)

AND

(Clinical Trial/ or ((before adj5 after) or (pre* adj5 post*) or intervention* or control* or trial* or random* or experiment* or time series or nonequivalent group* or effectiveness or ((follow* or adhere*) adj5 (recommendation* or advice))).ab,kw,ti.)

Medline

((exp aging/ or exp aged/ or frail elderly/ or geriatrics/ or cognitive dysfunction/ or multimorbidity/ or (older person? or older patient? or (senior* not school) or elder or elders or elderly or older adult* or geriatric* or cognitive dysfunction or multimorbidity or frailty or postmenopausal women or community-dwelling or (old* adj3 people) or (old* adj3 person?) or (old* adj3 patient?) or (old* adj3 client?)).ab,kf,ti. or (older m?n or older wom?n).ab,kf,ti. or (("65" or "70" or "75" or "80") adj2 (year? or yr* or age?)).ab,kf,ti. or (geriatr* or age or aging or elderl* or gerontolog*).jw.)

AND

(accidental falls/ or exp urinary incontinence/ or exp delirium/ or pressure ulcer/ or medication reconciliation/ or exp polypharmacy/ or medication therapy management/ or geriatric assessment/ or hospital planning/ or patient discharge/ or patient discharge summaries/ or (fall? or fell or falling or fallen or faller* or functional decline or delirium or urinary Incontinence or pressure ulcer* or polypharmacy or (review adj3 (drug? or medication)) or inappropriate medication* or medication reconciliation or medication changes or planning or geriatric assessment or discharge letter or discharge planning or discharge communication or (manag* adj3 therapy)).ab,kf,ti.))

AND

(exp university hospital/ or exp public hospital/ or exp hospital/ or exp hospital information system/ or or exp general hospital/ or exp hospital medicine/ or exp teaching hospital/ or exp hospital department/ or exp private hospital/ or (inpatient* or hospital* or ward* or ICU* or department of veterans affairs or intensive care unit* or specialist* or acute aged care setting or specialty or secondary care or tertiary care or hospital* or medical center*).ab,kf,ti.)

AND

("Decision Support Systems, Clinical"/ or (decision support or DSS or CDSS or CDS or CCDS or CCDSS or computer-generated or decision making support or expert system* or ((computer* or software or electronic or automat* or IT system* or information technology) and (tailor* or advice or advise or warning* or prompt* or remind* or alert* or algorithm* or tool* or order entry or order set*)) or computer* dos*3 or electronic dos*3 or automat* dos*3 or dos*3 calculator).ab,kf,ti.)

AND

(Interrupted Time Series Analysis/ or Controlled Before-After Studies/ or exp Clinical Trial/ or Historically Controlled Study/ or ((before adj5 after) or (pre* adj5 post*) or intervention* or control* or trial* or random* or experiment* or time series or nonequivalent group* or effectiveness or ((follow* or adhere*) adj5 (recommendation* or advice))).ab,kf,ti.)

Scopus

(TITLE-ABS-KEY ( "academic medical cent*" OR “hospital*” OR "length of stay" OR readmission OR “inpatient?” OR "critical care" OR "intensive care" OR icu OR "geriatric clinic" )) AND (( TITLE-ABS-KEY( "older person?" OR "older patient?" OR “senior*” OR elder OR elders OR elderly OR “geriatric*” OR “cognitive dysfunction” OR “multimorbidity” OR frailty OR "postmenopausal women" OR "community-dwelling" OR "old* people" OR "old* person?" OR "old* patient?" OR "old* client?" OR ( "older m*n" OR "older wom*n" ) OR ( ( "65" OR "70" OR "75" OR "80" ) W/1 ( “year*” OR yr* OR age* ) ) )) AND (TITLE-ABS-KEY("fall?" OR fell OR falling OR fallen OR "faller*" OR "functional decline" OR delirium OR "urinary incontinence" OR "pressure ulcer*" OR polypharmacy OR (“review” W/3 ("drug?" OR "medication")) OR "inappropriate medication*" OR "medication reconciliation" OR "medication changes" OR planning OR "geriatric assessment" OR "discharge letter" OR "discharge planning" OR "discharge communication" OR ("manag*" W/3 "therapy")))) AND ( TITLE-ABS-KEY ( "decision support" OR dss OR cdss OR cds OR ccds OR ccdss OR “computer-generated” OR "decision making support" OR "expert system*" OR "on-screen reminder*" OR ( (“computer*” OR software OR electronic OR “automat*” OR “it-system*” OR "information technology" ) AND (“tailor*” OR advice OR advise OR “warning*” OR “prompt*” OR “remind*” OR “alert*” OR “algorithm*” OR “tool*” OR "order entry" OR "order set*" ) ) OR "computer* dos*" OR "electronic dos*" OR "automat* dos*" OR "dos* calculator" ) ) AND ( TITLE-ABS-KEY ( ( ( before ) W/5 ( after ) ) OR ( ( “pre*” ) W/5 (“ post*” ) ) OR “*intervention*” OR “control*” OR “trial*” OR “random*” OR “experiment*” OR "time series" OR "nonequivalent group*" OR effectiveness OR ( ( “follow*” OR “adhere*” ) W/5 ( “recommendation*” OR advice ) ) ) )
