# Supplementary material for: Effect of Interventions With a Clinical Decision Support System for Hospitalized Older Patients: Systematic Review Mapping Implementation and Design Factors
Source: JMIR Med Inform. 2021 Jul 16;9(7):e28023. doi: 10.2196/28023 (PMC8325084; doi:10.2196/28023)
Supplement: Multimedia Appendix 3 [file medinform_v9i7e28023_app3.docx]

**Appendix 3: Study design, characteristics and outcomes of the included studies**

| Table 1: Study design, characteristics and outcomes of the included studies | | | | | |
| --- | --- | --- | --- | --- | --- |
| Author | Setting | Patients (nr), mean age, female (%) | Process outcomes (p-value) | Patient related outcomes | Other |
| Peterson et al. (2005)[42] | US  1 hospital | C: 1925, 74.6, 52.7  I: 1793, 74.8, 52.9 | * Agreement with recommendations:  ** All classes: C=18.6%, I=29.3%, (<0.001)  ** Benzodiazepines: C=20.8%, I=28.2%, (<0.001)  ** Opiates: C=16.6%, I=29.0%, (<0.001)  ** Neuroleptics: C=22.5%, I=38.0%, (<0.001)  ** Every class of psychotropic drugs: C=19.4%, I=29.3% (0.001)  * Orders with 10-fold dosing:  ** All classes: C=5.0%, I=2.8%, (<0.001)  ** Benzodiazepines: C=3.5%, I=2.0%, (0.01)  ** Opiates: C=5.5%, I=2.8%, (<0.001)  ** Neuroleptics: C=10.0%, I=7.5%, (0.35)  * Orders for non-recommended drugs: C=10.8%, I=7.6%, (<0.001)  * Drug orders written “to be giving as needed”: C=95%, I=82%, (<0.001) | * Altered mental status days per 100 patient-days: C=20.9, I=21.9, (0.17)  * Median LOS: C=4, I=4, (0.43)  * Falls per 100 patient-days rate: C=0.64, I=0.28, (0.001)  * Fall injuries per 100 patient-days rate: C=0.17, I=0.06, (0.09) | - |
| Terrell  et al. (2009)[32] | US  1 hospital | I: 2647, 73.5, 64.9  C: 2515, 73.7, 65  Number of visits | * Visits with an inappropriate medication prescription: C=3.9%, I=2.6%, (0.02)  * Prescriptions that were inappropriate: C=5.4%, I=3.4%, (0.006) | - | - |
| Dykes  et al. (2010)[37] | US  4 hospitals | 65+  C**:** 2509, -, -  I: 2755, -, - | - | Patient-days  * Fall rate: C=4.75, I=2.66, (0.003)  * Falls with injury: C=9, I=7, (0.66) | - |
| Malone  et al. (2010)[43] | US  3 units, 1 hospital | B: 478, -, -  A: 406, -, - | Percentage of patients  * Urinary catheter: B=26.2%, A=20.1%, (0.03)  * Physical therapy: B=27.0%, A=39.1%, (<0.001)  * Restraints: B=0.6%, A=0.8%, (>0.99)  * High risk medication: B=4.4%, A=4.0%, (0.78)  * Social work evaluation: B=86.0%, A=83.2%, (0.25) | Percentage of patients  * Pressure ulcer: B=7.9%, A=5.0%, (0.11)  * 30-day readmission: B=14.5%, A=12.7%, (0.40)  * Mean LOS (days): B=3.7, A=3.6, (0.76) | - |
| Holroyd-Leduc  et al. (2010)[36] | Canada  2 wards, 2 hospitals | B: 70, -, -  A: 64, -, - | - | Percentage of patients  * Delirium rate: Pre=33%, Post=31%, (0.840)  * Falls: Pre=10%, Post=6%, (0.430)  * Discharge to long-term care: Pre=13%, Post =6%, (0.200)  * Median LOS: Pre=14, Post=12, (0.740)  * Hospital readmission within 30 days: Pre=2, Post=4 (0.34) | - |
| Groshaus et al. (2012)[41] | Canada  6 units, 2 hospitals | - | * Order set use (estimated mean rate): 3.1 (95% 1.9-5.3) sets  higher after than before implementation  * Number of consults: B=278, A=262; (<0.2) | * Falls (estimated odds of a fall): 9.3 times higher before than after (0.065)  * Median LOS (days): B=42, A=45 (0.67) |  |
| Boustani  et al. (2012)[30] | US  1 hospital | I: 225, 76.8, 60.3  C: 199, 77.6, 71.1 | Percentage of patients  * Order ACE consults (first 48h): C=36%, I=42%, (0.40)  * Order ACE consults (hospital stay): C=49%, I=56%, (0.28)  * Order to discontinue FC (first 48h): C=18.9%, I=22.5%, (0.70)  * Order to discontinue FC (hospital stay): C=64.6%, I=61.7%, (0.86)  * Order to discontinue restraints (first 48h): C=0%, I=0%, (1.00)  * Order to discontinue restraints (hospital stay): C=0%, I=4.8%, (0.86)  * Order to discontinue AC (first 48h): C=7.4%, I=3.0%, (0.46)  * Order to discontinue AC (hospital stay): C=31.2%, I=48.9%, (0.11)  * Order FC (first 48h): C=23.6%, I=20.1%, (0.50)  * Order FC (hospital stay): C=35.1%, I=30.2%, (0.37)  * Order restraints (first 48h): C=3,6%, I=6.0%, (0.51)  * Order restraints (hospital stay): C=7,6%, I=10.5%, (0.66)  * Order AC (first 48h): C=14.7%, I=13.6%, (0.91)  * Order AC (hospital stay): C=21.3%, I=23.6%, (0.33) | * Mean LOS (days): C=6.8, I=7.7, (0.12)  Percentage of patients:  * Died within 30 days hospitalization: C=5.8%, I=6%, (0.69)  * Discharged home: C=36.9%, I=43.2%, (0.13)  * 30-day readmission: C=16.4%, I=18.6%, (0.53)  * At least one complication: C=44.9%, I=47.2%, (0.94)  ** Delirium: C=31.1%, I=33.7%, (0.78)  ** Pressure ulcer at discharge: C=11.1%, I=12.1%, (0.77)  ** Fall or injury at discharge: C=4.9%, I=4.5%, (0.88)  ** Physical restrained: C: 7.6%, I:11.1%, (0.54) | - |
| Khan  et al. (2013)[31] | US  1 hospital | I: 30, 74.2, 57  C: 30, 75.1, 47 | Percentage of patients  * Orders for geriatrician consult: C=40%, I=33%, (0.79)  * Order to discontinue restraints: C=0%, I=12%, (0.47)  * Order to discontinue urinary catheter: C=76%, I=72%, (0.99)  * Order to discontinue anticholinergic drugs: C=36%, I=67%, (0.37) | Percentage of patients  * Incidence delirium: C=29%, I=27%, (0.85)  * Incidence delirium ICU: C=12%, I=18%, (0.64)  * In-hospital mortality: C=17%, I=7%, (0.42)  * Survived 30 days after discharge: C=83%, I=87%, (0.99)  * Discharged home: C=30%, I=37%, (0.78)  * Mean LOS (days) ICU: C=5.7, I=7.4, (0.71)  * Mean LOS (days): C=12.2, I=14.5, (0.60) | - |
| Ghibelli  et al (2013)[46] | Italy  1 ward, academic hospital | I: 60, 81.1, 58 | * At least 1 PIM (percentage of patients): admission = 41.7%, discharge = 11.6%, (<0.001)  * Mean number of PIMs per patient: admission = 0.5, discharge = 0.1 (.001)  * At least 1 potentially severe DDI (percentage of patients): admission = 45%, discharge = 33.3%, (0.703)  * Number of new onset potentially severe DDI: admission = 59%, discharge = 33%, (<0.001) | - | - |
| Gurwitz  et al. (2014)[33] | US  1 hospital (part of group practice) | I: 1870, 79.0, 52.9  C: 1791, 79.1, 52  Number of discharges | Percentage of discharges  * Office visit 7 days: I=27.7%, C=28.3%, HR=0.95, (95%, 0.83, 1.1)  * Office visit 14 days: I=52.9%, C=52.5%, HR=0.98, (95%, 0.89, 1.1)  * Office visit 30 days: I=68.6%, C=68.8, HR=0.99, (95%, 0.91, 1.1) | Percentage of discharges  * Rehospitalization within 30 days: I=18.8%, C=19.9%, HR=0.94, (95% 0.81, 1.1) | - |
| Mattison  et al. (2014)[39] | US  1 hospital | B(c): 4919, 74.5, 47.4  B: 5077, 85.6, 57.8  A(c): 4482, 74.5, 48.5  A: 5571, 86.1, 58.2 | Frequency of orders  * Morphine: OR=0.52, (<0.001)  * Haloperidol: OR=0.6, (0.02)  * Trigger acute change in conscious state: OR=1.23, (0.49)  * Any trigger: OR=1.04, (0.71)  11* Trigger for marked RN concern: OR=0.85, (0.22) | * IC stay: OR=0.95, (0.62)  * In-hospital mortality: OR=1.19, (0.39)  * Discharged home: OR=1.18, (0.01)  * 3-day readmission: OR=1.02, (0.90)  * 30-day readmission: OR=1.16, (0.11)  * Mean LOS: OR=-0.04, (0.75) | - |
| O’Sullivan  et al. (2014)[25] | Ireland  1 hospital | T: 361, 77, 50 | * MAI score (number of medications): admission=15, follow-up=12, (0.001)  * ACOVE criteria (percentage of patients): admission=28.3%, follow-up=26.9%, (0.739)  * Prevalence PIP (percentage of patients)  ** STOPP: admission=64.2%, follow-up=55.5%, (<0.001)  ** Beers ID: admission=21%, follow-up=18.3%, (<0.05)  ** Beers CD: admission=31.8%, follow-up=31.6%, (0.282)  ** Priscus: admission=42.4%, follow-up=40.6%, (0.421)  ** Total: admission=76.3%, follow-up=71.2%, (<0.001)  * Prevalence PPO START: admission=31%, follow-up=31.5%, (0.512) | - | - |
| O'Sullivan et al. (2016)[26] | Ireland  1 hospital | I: 361, 77, 50  C: 376, 78, 50 | - | * ADR (proportion of patients): I=13.9%, C=20,7%, (0.02)  (Adjusted p=.01)  * Median LOS (days): I=8, C=9, (0.444)  * All-cause mortality rate: I=4.7%, C=4.5% (no significant difference) | - |
| Gallagher et al*.* (2016)[27] | Ireland  1 hospital | I: 361, 77, 50  C: 376, 78, 50 | - | * ADR events difference in mean: -0.064, (0.081)  * ADR event: OR=0.655, (0.047) | * Costs difference in mean: - 807, (0.548) |
| Stevens  et al. (2015)[29] | US  1 VAMC | T**:** 4042, -, - | * Average monthly PIMs prescribed (percentage of prescriptions): B=9.4%, A=4.6%, (<0.001) | - | - |
| Stevens  et al. (2017)[28] | US  4 VAMCs | - | * Average monthly PIMs prescribed (percentage of prescriptions):  ** Site 1: B=11.9%, A=5.1%, (<0.0001)  ** Site 2: B=8.2%, A=4.5%, (<0.0001)  ** Site 3: B=8.9%, A=6.1%, (0.0007)  ** Site 4: B=7.4%, A=5.7%, (0.04) | - | - |
| Cossette et al. (2016)[35] | Canada  1 hospital, 2 sites | T: 8622, 83.3, 54.5 | Rate of patient-days with at least one PIM  * Immediately after intervention:  ** Most parsimonious model: -3.45% (-4.76  to – 2.14, p<.001)  ** Full model: -2.55%  (-5.58 to 0.47, p=.12)  * Per month post intervention period:  ** Full model: +0.11%  (-0.40 to 0.62, p=.68)  Gastrointestinal agents  * Immediately after intervention:  ** Most parsimonious model: -1.61% (-2.14  to – 1.07, p<.001)  ** Full model: -1.20%  (-2.72 to 0.31, p=.14)  * Per month post intervention period:  ** Full model: -0.09  (-0.34 to 0.15, p=.47)  Dimenhydrinate  * Immediately after intervention:  ** Most parsimonious model: -7.07% (-9.19  to – 4.96, p<.001)  ** Full model: -6.51%  (-8.98 to -4.05, p<.001)  * Per month post intervention period:  ** Full model: -0.17%  (-0.57 to 0.22 p=.40)  Ondansetron  * Immediately after intervention:  ** Most parsimonious model: 8.99% (7.23  to 10.75, p<.001)  ** Full model: 8.99% (7.23 to 10.75, p<.001)  * Per month post intervention period:  ** Full model: -0.34%  (-0.63 to -0.05 p=.03) | - | - |
| Cossette et al. (2017)[34] | Canada  1 hospital,  2 sites | I: 126, 81.5, 61.9  C: 128, 80.5, 58.6 | * Absolute difference drug cessation/dosage decrease at 48h: +30.0%, (13.8 – 46.1)  * Absolute difference drug cessation/dosage decrease at 15discharge: +20.8%, (4.6 – 37.0)  * Difference drug cessation/dosage decrease (regardless of clinical relevance) at 48h post-alert: +16.2%, (95% 2.9-29.6)  * Difference drug cessation/dosage decrease (regardless of clinical relevance) at discharge: +8.0%, (95% -4.0 to 20.0) | * Median LOS: C=9.5, I=10, (0.9)  * In-hospital deaths (n): C=11, I=6, (0.3)  * 30-day post-discharge ER visits (n): C=27, I=27, (1.0)  * 30-day post-discharge readmissions (n): C=28, I=20, (0.3) | - |
| Lagrange et al. (2017)[38] | France  9 health-care institu-tions | B: 185, 75.4, 63  A: 187, 73.9, 63 | Delivered drug doses  * Antipsychotics: +21%  * Neuroleptics: +11%  * Short-acting (<20 h) benzodiazepines: +47%  * Long-acting (>20 h) benzodiazepines: 0%  * Anticholinergics: -28%  * Antiparkinson:+66%  * Statins: +22%  * PPI: +49% | * Mean LOS (days): +12%  * Falls (n): -40% | - |
| Adeola  et al. (2018)[40] | US  tertiary care facility and 4 community hospitals | B**:** 21541, 79.8, -  A: 27764, 79.8, -  hospital admission | * Proportion hospital admissions with at least 1 of the target medications: B=45.6%, A=31.3%, RR=31.4%  ** Lorazepam: B=13.3%, A=12.3%, (<0.001)  ** Zolpidem: B=11.2%, A=5.3%, (<0.001)  ** Diphenhydramine: B=12.9%, A=7.1%, (<0.001)  ** Methocarbamol: B=3.2%, A=2.8%, (0.008)  ** Hydroxyzine: B=1.3%, A=0.7%, (<0.001)  ** Diazepam: B=1.5%, A=1.6%, (0.19)  ** Cyclobenzaprine: B=1.2%, A=1.1%, (0.04)  ** Carisoprodol: B=0.3%, A=0.2%, (0.07)  ** Meperidine: B=0.6%, A=0.2%, (<0.001)  * Mean number of doses of target medications  ** Lorazepam: B=4.2, A=3.6, (<0.001)  ** Zolpidem: B=3.4, A=2.9, (<0.001)  ** Diphenhydramine: B=2.5, A=2.1, (<0.001)  ** Methocarbamol: B=7.4, A=7.4, (0.93)  ** Hydroxyzine: B=5.0, A=4.5, (0.43)  ** Diazepam: B=3.7, A=4.1, (0.36)  ** Cyclobenzaprine: B=4.4, A=3.1, (0.008)  ** Carisoprodol: B=5.7, A=11.8, (0.04)  ** Meperidine: B=3.2, A=2.5, (0.31)  * Average dose of target medications (mg)  ** Lorazepam: B=0.9, A=0.8, (0.75)  ** Zolpidem: B=6.2, A=5.3, (<0.001)  ** Diphenhydramine: B=23.3, A=22.9, (0.08)  ** Methocarbamol: B=645.5, A=577.4, (<0.001)  ** Hydroxyzine: B=24.6, A=22.0, (0.006)  ** Diazepam: B=4.9, A=4.1, (<0.001)  ** Cyclobenzaprine: B=8.5, A=7.1, (<0.001)  ** Carisoprodol: B=338.6, A=323.6, (0.13)  ** Meperidine: B=34.5, A=27.3, (0.02)  * maximum dose of target medications:  ** Lorazepam: B=0.9, A=0.9, (0.59)  ** Zolpidem: B=6.3, A=5.4, (<0.001)  ** Diphenhydramine: B=24.5, A=23.9, (0.03)  ** Methocarbamol: B=658.9, A=598.9, (<0.001)  ** Hydroxyzine: B=25.1, A=22.7, (0.01)  ** Diazepam: B=5.1, A=4.3, (<0.001)  ** Cyclobenzaprine: B=8.5, A=7.3, (<0.001)  ** Carisoprodol: B=340.2, A=328.1, (0.20)  ** Meperidine: B=36.5, A=28.4, (0.01) |  | - |
| Booth  et al. (2019)[44] | US  2 units, 1 hospital | B: 48, 74, 71  A; 113, 74, 54 | Completed assessment:  * Current Katz: B=62.5%, A=88.5% (<.001)  * Nu-DESC: B=4.2%, A=96.5% (<.001)  * Baseline Kat: B=60.4%, A=64.6% (>.001)  * SIS: B=89.6%, A=94.7% (>.001)  Mobility:  * Up to chair B=36.4%, A=63.5% (.04)  * Walked in hall: B= 18.2%, A=33.7% (>.05)  * Walked off unit: B=0%, A=1.2% (>.05)  * Prevalence positive Nu-DESC score:  B=13.6%, A=4.8% (.16)  * Case-mix index B= 2.43, A= 2.39 (0.90) | * Mean LOS: B=4.9, A=4.3 (0.38) | * Variable cost B= $12,466, A= $11,365 (0.54) |
| McDonald et al. (2019)[45] | Canada  3 hospitals | B: 383, 79, 53  A: 417, 81, 53 | Proportion of patients with ≥ 1 PIM and deprescription at discharge: B:46.9%, A:54.7% (OR = 1.4, CI=1.1-1.8) | Occurrence of adverse events within 30 days of discharge: 35.8 (74/207). A: 32.8% (76/232) p=.51  Occurrence of ADEs within 30 days of discharge: B=5.2%(10/192), A=5%(11/218) p=.94 | - |

A = After; AC = AntiCholinergic medications; ACE = Acute Care for Elders; ACOVE = Assessment of Care Of Vulnerable Elders; ADR = Adverse Drug Reaction; DDI = Drug–Drug Interaction; FC = Foley Catheterization; LOS = Length Of Stay; MAI = Medication Appropriateness Index; PIM = Potential Inappropriate Medication; PIP = Potential Inappropriate Prescription; PPI = Proton Pump Inhibitors; PPO = Potential Prescribing Omissions; RCT = Randomized Controlled Trial; VAMC = Veteran Affairs Medical Center

Outcome with underscore = primary outcome
