# Supplementary material for: Effect of Interventions With a Clinical Decision Support System for Hospitalized Older Patients: Systematic Review Mapping Implementation and Design Factors
Source: JMIR Med Inform. 2021 Jul 16;9(7):e28023. doi: 10.2196/28023 (PMC8325084; doi:10.2196/28023)
Supplement: Multimedia Appendix 4 [file medinform_v9i7e28023_app4.docx]

**Appendix 4. Study design, characteristics, outcomes, and main implementation or design factors of the included articles.**

| Table 1: Study design, characteristics, outcomes, and main implementation or design factors of the included articles. | | | | | | | | | | | | | | |
| --- | --- | --- | --- | --- | --- | --- | --- | --- | --- | --- | --- | --- | --- | --- |
| I | Author | Geriatric topic | | | | | | Study design | Patients | Outcomes | Effect | Implementa-tion/ design factors | | |
|  |  | D | D/A | F | FD | M | P |  |  |  |  | I1 | I2 | D1 |
| 1 | Peterson et al [42] | N/A^a^ | N/A | ✓^b^ | N/A | N/A | N/A | Before-after | Control: 1925; Intervention: 1793 | Process; patient | Yes | −^c^ | − | +^d^ |
| 2 | Terrell et al [32] | N/A | ✓ | N/A | N/A | N/A | N/A | RCT^e^ | Intervention: 2647; control: 2515 | Process | Yes | + | − | + |
| 3 | Dykes et al [37] | N/A | N/A | ✓ | N/A | N/A | N/A | Cluster-randomized study | Control: 2509; intervention: 2755 | Patient | Yes | + | − | + |
| 4 | Malone et al [43] | N/A | N/A | N/A | ✓ | N/A | ✓ | Before-after | Before: 478; after: 406 | Process; patient | No | − | + | − |
| 5 | Holroyd-Leduc et al [36] | ✓ | N/A | ✓ | N/A | N/A | N/A | ITS^f^ | Before: 70; after: 64 | Patient | No | − | − | + |
| 6 | Groshaus et al [41] | ✓ | N/A | ✓ | ✓ | N/A | N/A | Stepped wedge trial | N/A | Process; patient | Yes | − | + | + |
| 7 | Boustani et al [30] | ✓ | N/A | ✓ | N/A | ✓ | ✓ | RCT | Intervention: 225; control: 199 | Process; patient | No | − | − | − |
|  | Khan et al [31] | ✓ | N/A | N/A | N/A | ✓ | N/A | RCT | Intervention: 30; control: 30 | Process; patient | No | − | − | − |
| 8 | Ghibelli et al [46] | N/A | N/A | N/A | N/A | ✓ | N/A | Before-after | Intervention: 60 | Process | Yes | − | − | − |
| 9 | Gurwitz et al [33] | N/A | ✓ | N/A | N/A | N/A | N/A | RCT | Intervention: 1870; control: 1791 | Process; patient | No | − | − | − |
| 10 | Mattison et al [39] | ✓ | N/A | N/A | N/A | N/A | N/A | Before-after with controls | Before (control): 4919; before: 5077; after (control): 4482; after: 5571 | Process; patient | Yes | + | + | + |
| 11 | O’Sullivan et al [25] | N/A | N/A | N/A | N/A | ✓ | N/A | Before-after | Total: 361 | Process | Yes | − | + | + |
|  | O’Sullivan et al [26] | N/A | N/A | N/A | N/A | ✓ | N/A | RCT | Intervention: 361; control: 376 | Patient | Yes | − | + | + |
|  | Gallagher et al [27] | N/A | N/A | N/A | N/A | ✓ | N/A | RCT^g^ | Intervention: 361; control: 376 | Patient; cost | Yes | − | + | + |
| 12 | Stevens et al [29] | N/A | ✓ | N/A | N/A | N/A | N/A | Before-after | Total: 4042 | Process | Yes | + | + | + |
|  | Stevens et al [28] | N/A | ✓ | N/A | N/A | N/A | N/A | Before-after | N/A | Process | Yes | + | + | + |
| 13 | Cossette et al [35] | ✓ | N/A | N/A | ✓ | ✓ | N/A | ITS | Total: 8622 | Process | Yes | + | + | − |
| 14 | Cossette et al [34] | ✓ | N/A | ✓ | ✓ | ✓ | N/A | RCT | Intervention: 126; control: 128 | Process; patient | Yes | + | + | − |
| 15 | Lagrange et al [38] | N/A | N/A | ✓ | N/A | ✓ | N/A | Before-after | Before: 185; after: 187 | Process | No | − | − | − |
| 16 | Adeola et al [40] | ✓ | N/A | N/A | N/A | ✓ | N/A | Before-after | Before: 21,541; after: 27,764 | Process | Yes | + | + | + |
| 17 | Booth et al [44] | ✓ | N/A | N/A | ✓ | N/A | N/A | Before-after | Before: 48; after: 113 | Process; patient; cost | Yes | + | + | + |
| 18 | McDonald et al [45] | N/A | ✓ | N/A | N/A | ✓ | N/A | Before-after | Before: 383; after: 417 | Process; patient | Yes | + | − | − |

D = Delirium; D/A = Discharge and aftercare; D1= Workflow; F = Falls; FD = Functional decline; I = Intervention; I1= A priori analysis; I2 = Multifaceted; M = Medication review; P = Pressure ulcer;

^a^N/A: not applicable.

^b^Geriatric topic described in article.

^c^Implementation or design factor not described in article.

^d^Implementation or design factor described in article.

^e^RCT: randomized controlled trial.

^f^ITS: interrupted time series.

^g^Cost-effectiveness.
