# Supplementary material for: Effect of Interventions With a Clinical Decision Support System for Hospitalized Older Patients: Systematic Review Mapping Implementation and Design Factors
Source: JMIR Med Inform. 2021 Jul 16;9(7):e28023. doi: 10.2196/28023 (PMC8325084; doi:10.2196/28023)
Supplement: Multimedia Appendix 5 [file medinform_v9i7e28023_app5.docx]

**Appendix 5: Risk of bias**

| Table 1: Risk of bias of RCTs and controlled before-after studies | | | | | | | | | |
| --- | --- | --- | --- | --- | --- | --- | --- | --- | --- |
|  | 1 | 2 | 3 | 4 | 5 | 6 | 7 | 8 | 9 |
| Peterson et al.  (2005) [42] | + | + | ? | - | ? | - | + | - | - |
| Terrell et al. (2009)[32] | - | - | - | - | ? | - | ? | - | - |
| Dykes et al. (2010)[37] | - | - | - | - | ? | - | ? | - | - |
| Malone et al. (2010)[43] | + | + | ? | ? | ? | ? | - | ? | + |
| Groshaus et al. (2012)[41] | + | + | ? | + | ? | - | ? | - | + |
| Boustani et al. (2012)[30] | - | - | - | - | ? | - | + | - | - |
| Khan et al. (2013)[31] | - | - | - | - | ? | - | + | - | - |
| Ghibelli et al (2013)[46] | + | + | + | - | ? | - | - | - | + |
| Gurwitz et al. (2014)[33] | - | - | - | - | ? | - | + | - | - |
| Mattison et al. (2014)[39] | + | + | ? | - | ? | - | - | - | - |
| O’Sullivan et al. (2014)[25] | + | + | ? | ? | ? | + | ? | - | - |
| O'Sullivan et al. (2016)[26] | + | - | - | - | ? | + | ? | - | - |
| Gallagher et al. (2016)[27] | + | - | - | - | ? | ? | ? | - | - |
| Stevens et al. (2015)[29] | + | + | ? | + | ? | ? | - | - | - |
| Stevens et al. (2017)[28] | + | + | ? | + | ? | ? | - | - | - |
| Cossette et al. (2017)[34] | - | - | + | - | ? | - | + | - | - |
| Lagrange et al. (2017)[38] | + | + | ? | ? | ? | ? | - | ? | - |
| Adeola et al. (2018)[40] | + | + | ? | ? | ? | - | - | - | - |
| Booth et al. (2019)[44] | + | + | ? | + | ? | ? | - | - | - |
| McDonald et al. (2019)[45] | + | + | ? | + | ? | - | - | - | - |

1 = Random sequence generation; 2 = Allocation concealment; 3 = Baseline outcome measurements similar; 4 = Baseline characteristics similar; 5 = Incomplete outcome data; 6 = Knowledge of the allocated interventions; 7 = Protection against contamination; 8 = Selective outcome reporting; 9 = Other risks of bias

+ High risk

? Unclear risk

- Low risk

Intermittent time series analysis studies

| Table 2: Risk of bias of RCTs and controlled before-after studies | | | | | | | |
| --- | --- | --- | --- | --- | --- | --- | --- |
|  | 1 | 2 | 3 | 4 | 5 | 6 | 7 |
| Holroyd-Leduc et al.  (2010)[36] | - | - | - | ? | ? | - | - |
| Cossette et al.  (2016)[35] | - | - | - | - | ? | - | - |

1 = Was the intervention independent of other changes?; 2 = Was the shape of the intervention effect pre-specified?; 3 = Was the intervention unlikely to affect data collection?; 4 = Was the knowledge of the allocated interventions; 5 = incomplete outcome data adequately addressed?; 6 = Was the study free from selective reporting?; 7 = Other risks of bias

+ High risk

? Unclear risk

- Low risk
