# Supplementary material for: Effect of Interventions With a Clinical Decision Support System for Hospitalized Older Patients: Systematic Review Mapping Implementation and Design Factors
Source: JMIR Med Inform. 2021 Jul 16;9(7):e28023. doi: 10.2196/28023 (PMC8325084; doi:10.2196/28023)
Supplement: Multimedia Appendix 6 [file medinform_v9i7e28023_app6.docx]

**Appendix 6: Implementation and design factors described in the included studies**

| Table 1: Implementation and design factors described in the included studies | | | | | | | | |
| --- | --- | --- | --- | --- | --- | --- | --- | --- |
| Author | Implementation factors | | | | Design factors | | | |
|  | Analysis problem ^a^ | Implementation strategies^b^ | | Development  integration  evaluation ^c^ | Content | Context | System | Imple-menta-tion |
|  |  | Multifaceted | Strategies |  |  |  |  |  |
| Peterson  et al. (2005)[42] | - | No | Reminders CDSS | - | Clinical knowledge Literature, drug monographs, geriatrician, geriatric psychiatrist, pharmacist, 2 internists, anesthe-siologist  Presenta-tion  Alert with suggestions for geriatric dosing, alert for substitution, reduced dose or frequency | Patient data  Age  Users Physicians  Clinical workflow Existing CDSS | Channel CDSS within order entry  Trigger Entering order | - |
| Terrell  et al. (2009)[32] | Expert panel reviewed previous year’s pre-scribing data | No | Reminders CDSS | - | Clinical knowledge Beers criteria, pharmacists, geriatricians, information technology experts, emergency physicians.  Presenta-tion  Alert with option to order/reject recom-mended alternative, a second menu displayed for reason of rejecting | Users Physicians  Clinical workflow CDSS fitted into the user’s workflow | Trigger Prescrip-tion of targeted drugs at discharge | - |
| Dykes  et al. (2010)[37] | Barriers and facilita-tors to fall risk communication and inter-ventions | No | RemindersCDSS | Integration Institute  for Healthcare Improve-ment’s Framework for Spread was used | Clinical knowledge Literature, findings from phase 1 interviews.  Presenta-tion  Tailored fall prevention interventions using icons to address patients’ fall risk, a tailored poster, patient education handout, and plan of care was printed after approval | Patient data  MFS risk factors  Users Nurses  Clinical workflow Existing commu-nication and workflow patterns | - | - |
| Malone  et al. (2010)[43] | - | Yes | Organisa-tional culture ACE advisory committee, ACE interdisci-plinary team  RemindersReport  Educa-tional  E-geriatrician information during meetings, instruction and discussion of approach regularly | Evaluation Number of reports, experiences, predictive values of the ACE tracker | Clinical knowledge Software program-mers, geriatricians, nurses, pharmacists, refinements during 24 months  Presenta-tion  Real time report with for example length of stay, cognitive impairment, bed rest, number of prescribed drugs, falls, pressure ulcer | Patient data  Data from various parts of the EMR  Users During ACE meeting | Trigger Updated every 15 minutes | Explana-tion use of the report during medical staff meetings |
| Holroyd-Leduc  et al. (2010)[36] | - | No | RemindersCDSS | Evaluation Adaption of care pathway based on needs focus group | Clinical knowledge Trials, CAM, information technolo-gists, decision makers, researchers, orthopedics, geriatrics and nursing  Presenta-tion  General delirium interventions into an order set | Users Nurses  Clinical workflow Before interven-tion, a post-operative hip fracture order set existed | Channel Hospitals' EMR | Barriers for using the pathway |
| Groshaus et al. (2012)[41] | - | Yes | RemindersCDSS  Educational Education about care for older patients, binder of geriatric resource materials | Integration MRC framework for complex interventions and Knowledge to Action Cycle were used.  Evaluation Barriers and facilitators CDSS implement-tation | Clinical knowledge Clinical trials, systematic reviews, guidelines addressing delirium, falls, continence, nutrition and hydration  Presenta-tion Electronic nurse-initiated order set | Users Nurses  Clinical workflow Workflow impact and  sustaina-bility were considered | Channel Order set within  hospitals’ EMR | * Approval of nurses, managers, local leaders, and informa-ticians.  * Educa-tional materials/ in-servicing for order set. |
| Boustani  et al. (2012)[30] | - | No | RemindersCDSS | - | Clinical knowledge Systematic reviews, guidelines, monthly discussions interdisci-plinary team for 1 year  Presenta-tion  * Non-interruptive alerts, quick exit using the F8 key  * Interrup-tive alerts, the alert required a decision to accept, reject, or modify | Users Physicians | Trigger Placement of an order in the CPOE | - |
| Khan  et al. (2013)[31] | - | No | See Boustani et al. (2012) | - | See Boustani et al. (2012) | See Boustani et al. (2012) | See Boustani et al. (2012) | - |
| Ghibelli  et al (2013)[46] | - | No | RemindersCDSS | - | Clinical knowledge Beers’ 2003 criteria, anticholi-nergic cognitive burden scale, potential DDIs, dose adjustment renal impairment, GerontoNet ADR Risk Score  Presenta-tion  Patient specific report including DDIs, PIMs. | Patient data Conditions,  drugs, previous ADR, renal failure | Channel Stand-alone system | - |
| Gurwitz  et al. (2014)[33] | - | No | RemindersCDSS | Development Physicians reviewed CDSS prior implementa-tion and suggested modifications | Clinical knowledge National advisory committee, local clinicians/ pharmacists selected high-risk drugs, informatics team used blueprints  Presenta-tion Information new drugs at hospital discharge, warnings DDIs, dose changes and monitoring, reminder to schedule a visit after discharge. | Patient data  Data on drugs from the EHR and health plan data  Users Primary care providers and staff | Channel In-basket message in EHR  Trigger  On day 3/ a week after hospital discharge | Before go-live,  information by memo |
| Mattison  et al. (2014)[39] | Before inter-vention, a standard bedside form was used | Yes | RemindersCDSS, bedside checklist for delirium prevention  Educational Sessions and emails during first 6 weeks | Development Geriatricians, hospitalists and nurses developed a supplemental checklist | Clinical knowledge Specialist at the institution Presenta-tion: Modified orders, dose recommen-dations | Patient data  Data from CPOE system  Users Physicians  Clinical workflow Before interven-tion, the CPOE included Beers criteria | Channel CPOE interface with decision support  Trigger Order | - |
| O’Sullivan  et al. (2014)[25] | - | Yes | See O’Sullivan et al. (2016) | - | See O’Sullivan et al. (2016) | See O’Sullivan et al. (2016) | See O’Sullivan et al. (2016) | - |
| O'Sullivan et al. (2016)[26] | - | Yes | Organisa-tional culture Medication reconcilia-tion, pharma-ceutical care plan  RemindersCDSS | - | Clinical knowledge START, STOPP, Beers criteria (v3) and PRISCUS criteria, British National Formulary. product characteris-tics data, MDRD formula and laboratory, collaboration between pharmacy and geriatric medicine  Presenta-tion  Patient specific recommen-dations, including DRPs and DDIs, review clinical relevance, written pharma-ceutical care plan for medical team | Patient data Structured data  Users Pharma-cist  Clinical workflow Before interven-tion, hospital pharma-cists did unstruck-tured reviews with hand-written notes and sometimes medication reconciliation | Channel Stand-alone system | - |
| Gallagher et al. (2016)[27] | - | Yes | See O’Sullivan et al. (2016) | - | See O’Sullivan et al. (2016) | See O’Sullivan et al. (2016) | See O’Sullivan et al. (2016) | - |
| Stevens  et al. (2015)[29] | CPOE mapping and inter-views | Yes | Educatio-nal  Geriatric pharma-cology lecture for ED providers  Reminders  * Reminder card top 5 PIMs  * CDSS  Audit & Feedback  One-to-one feedback | Development The Vision-Analysis-Team-Aim-Map-Measure-Change-Sustain model of process improvement was used | Clinical knowledge Beers criteria, ED physicians, geriatricians, gerontolo-gists, pharmacists, nurses, applications coordinators  Presenta-tion  Dose adjust-ments, prepopu-lated fields, point of prescribing education, links to geriatric content in order sets. | Users Physicians  Clinical workflow Integrated into existing order sets | Channel Templates in EMR | Pharma-cists, ED providers, the local pharma-cy and thera-peutics com-mittee reviewed before imple-menta-tion |
| Stevens  et al. (2017)[28] | See Stevens et al. (2015) | Yes | See Stevens et al. (2015) | See Stevens et al. (2015) | See Stevens et al. (2015)  Additional: Passive drug warnings at point of prescribing designed to minimize alert fatigue | See Stevens et al. (2015) | See Stevens et al. (2015) | See Stevens et al. (2015) |
| Cossette et al. (2016)[35] | Prescrip-tion data  reviewed | Yes | RemindersCDSS  Practice and setting Inpatient geriatric consulta-tion team  Educatio-nal  List with PIMs was emailed and presented for physicians and pharma-cists (customi-zed to each specialty) | Development  Pharmacy and therapeutics committee approved the PIM list  Integration  * Implemen-tation KT strategy  * Clinicians’ opinions and barriers were discussed  * Presentations were modified based on feedback | Clinical knowledge Beers’ 2012 criteria, prescription data, expert committee (geriatrics, pharmacy, internal medicine) selected PIMs related to delirium and functional decline.  Presenta-tion  Alerts to target PIMs and drug–disease and drug–syndrome interactions, reviewed and developed a plan for the physician | Patient data Active drugs as proxies for dementia and parkin-son’s disease or parkinso-nism  Users Pharma-cists | Channel Stand-alone system, generation of alerts each night | Review of clinical rele-vance of the alerts. |
| Cossette et al. (2017)[34] | See Cossette et al. (2016) | Yes | See Cossette et al. (2016) | See Cossette et al. (2016) | Clinical knowledge Beers and STOPP criteria, expert panel (geriatric-cians, internists, and pharmacists) selected geriatric explicit criteria  Presenta-tion  Alerts were evaluated for clinical relevance, and a plan was developed for the treating physician | Patient data Hospital’s EMR, hospital-coded discharge summary, active drugs as proxies for dementia and parkin-son’s disease or parkin-sonism  Users Pharma-cists | Channel See Cossette et al (2016)  Trigger Drugs adapted <48 h. (acute delirium sooner for fast medication review) | - |
| Lagrange et al. (2017)[38] | - | No | RemindersCDSS | - | Clinical knowledge  Guidelines, practice indicators  Presenta-tion:  Patient-specific report with PIMs and alternatives, dose/drug adaptions, disorders, adherence, compliance with indicators, report could be exported and used to write a systematic report | Patient data  Last prescript-tions (data in patient file)  Users Pharma-cists | Channel Stand-alone system, 45 sec | - |
| Adeola  et al. (2018)[40] | Assess-ment of clinical alerts | Yes | Audit & Feedback Peer-to-peer feedback, daily com-pliance report  RemindersCDSS  Local consensus processes Formulary and policy changes  Educa-tional Posters, brochures/videos,  presentations, self-study modules, daily emails, news-letters | Evaluation Successful reduction of target medications, some were initiated/ resumed at discharge | Clinical knowledge Beer’s 2012 criteria, clinical pharmacists and geriatricians selected drugs possible associated with delirium  Presenta-tion  * Clinical alerts for review with several possible actions: dose reduction, disconti-nuation, switch or continuation.  * Best practice warning alerts during discharge medication reconcilia-tion  * Age-conditional order set modifica-tions | Patient data  Active orders of target drugs  Users Physicians and pharma-cists  Clinical workflow Before interven-tion, target drugs were prescribed through order sets | Channel EPS software generated alerts | Approval of 30 commit-tees for changes in order sets |
| Booth  et al. (2019)[44] | Report (ACE tracker) was already in use | Yes | Reminders  * Algo-rithms for non-pharma-cologic manage-ment of pain, mobidity, delirium (virtual ACE)  * Orderset  Educa-tional  * Teaching on screening assess-ment, ACE tracker and algorithms.  * Coach (geriatric nurse coordina-tor) providing feedback  * Training | Integration Embedded 3 geriatric screening assessments in EMR (Katz index, SIS, Nu-DESC)  * Workflow 10 min extra time | Presenta-tion  Unit based report identifying older patients at risk for adverse outcomes (delirium, functional decline) | Users  Nurses, Patient care techni-cians, secretaries, rehabilita-tion therapists, dieticians, pharma-cists, case managers, social workers, orthopedic residents, hospital physicians  Clinical workflow Existing order sets were modified | Channel  * EMR (order sets)  * Stand-alone (algo-rithms) | Staff engage-ment and training on ACE tracker and algo-rithms |
| McDonald et al. (2019)[45] | Usual care:  medica-tion recon-ciliation | No | Reminder CDSS report from MedSafer | Development  Rules converted by expert physicians with background in healthcare informatics. | Clinical knowledge  Beers criteria, STOPP, Choosing wisely list. Teams of physicians/ pharmacists  Presenta-tion  Patient specific depre-scribing report. Patients received brochure on poly-pharmacy harms and depre-scribing | Patient data  Medical and medication history (typed in MedSafer) | Channel Stand-alone system | Clinical teaching unit teams inter-views monthly for false positive and negative recom-menda-tions |

ACE = Acute Care for Elders; ADR = Adverse Drug Reactions; CAM = Confusion Assessment Method; CDSS = Clinical Decision Support System; CPOE = Computerized Physician Order Entry; DDI = Drug drug interactions; ED = Emergency Department; EHR = Electronic Health Record; EMR = Electronic Medical Record; EPS = Electronic Pharmacy Surveillance; I = Intervention; MDRD = Modification of Diet in Renal Disease; MFS = Morse Falls Scale; PIM = Potential Inappropriate Medication

^a^ Step 1 (development of proposal for change), 2 (analysis of actual performance, targets for charge) and 3 (problem analysis of target group and setting) of the Grol and Wensing Implementation of Change Model

^b^ Step 4 of the Grol and Wensing Implementation of Change model and classification of implementation strategies of the EPOC taxonomy

^c^ Step 5, 6 and 7 of the Grol and Wensing Implementation of Change model
